# Supplementary material for: A recombinant human immunoglobulin with coherent avidity to hepatitis B virus surface antigens of various viral genotypes and clinical mutants
Source: PLoS One. 2020 Aug 13;15(8):e0236704. doi: 10.1371/journal.pone.0236704 (PMC7425877; doi:10.1371/journal.pone.0236704)
Supplement: S1 Raw images — (PDF) [file pone.0236704.s001.pdf]

Supporting Information 1

	1 10	20	30	40	50	60	70	80	90	100	110	120
A1	MENITSGFLG	PLLVLQAGFF	LLTRILTIPQ	SLDSWWTSLN	FLGGSPVCLG	QNSQSPTSNH	SPTSCPPICP	GYRWMCLRRF	IIFLFILLLC	LIFLLVLLDY	QGMLPVCPLI	PGSTTTSTGP
A2	MENITSGFLG	PLLVLQAGFF	LLTRILTIPQ	SLDSWWTSLN	FLGGAPVCLG	QNSQSPTSNH	SPTSCPPICP	GYRWMCLRRF	IIFLFILLLC	LIFLLVLLDY	QGMLPVCPLI	PGSTTTSTGP
B1	MENIASGLLG	PLLVLQAGFF	LLTKILTIPQ	SLDSWWTSLN	FLGGTPVCLG	QNSQSQISSH	SPTCCPPICP	GYRWMCLRRF	IIFLCILLLC	LIFLLVLLDY	QGMLPVCPLI	PGSSTTSTGP
B2	MENIASGLLG	PLLVLQAGFF	SLTKILTIPQ	SLDSWWTSLN	FLGETPVCLG	QNSQSQISSH	SPTCCPPICP	GYRWMCLRRF	IIFLCILLLC	LIFLLVLLDY	QGMLPVCPLI	PGSSTTSTGP
C1	MENTTSGFLG	PLLVLQAGFF	LLTRILTIPQ	SLDSWWTSLN	FLGGAPTCPG	QNSQSPTSNH	SPTSCPPICP	GYRWMCLRRF	IIFLFILLLC	LIFLLVLLDY	QGMLPVCPLL	PGTSTTSTGP
C2	MESTTSGFLG	PLLVLQAGFF	LLTRILTIPQ	SLDSWWTSLN	FLGGAPACPG	QNLQSPTSNH	SPTSCPPICP	GYRWMCLRRF	IIFLFILLLC	LIFLLVLLDY	QGMLPVCPLL	PGTSTTSTGP
D1	MENITSGFLG	PLLVLQAGFF	LLTRILTIPQ	SLDSWWTSLN	FLGGTTVCLG	QNSQSPTSNH	SPTSCPPTCP	GYRWMCLRRF	IIFLFILLLC	LIFLLVLLDY	QGMLPVCPLI	PGSSTTSTGP
D2	MENITSGFLG	PLLVLQAGFF	LLTRILTIPQ	SLDSWWTSLN	FLGGTTVCLG	QNSQSPTSNH	SPTSCPPTCP	GYRWMCLRRF	IIFLFILLLC	LIFLLVLLDY	QGMLPVCPLI	PGSSTTSVGP
E1	MESITSGFLG	PLLVLQAGFF	LLTKILTIPQ	SLDSWWTSLN	FLGGAPVCLG	QNSQSPTSNH	SPTSCPPICP	GYRWMCLRRF	IIFLFILLLC	LIFLLVLLDY	QGMLPVCPLI	PGSSTTSTGP
E2	MEGITSGFLG	PLLVLQAGFF	LLTKILTIPQ	SLDSWWTSLN	FLGGAPVCLG	QNSQSPISNH	SPTSCPPICP	GYRWMCLRRF	IIFLFILLLC	LIFLLVLLDY	QGMLPVCPLI	PGSSTTSTGP
F1	MDNITSGLLG	PLLVLQAVCF	LLTKILTIPQ	SLDSWWTSLN	FLGGLPGCPG	QNSQSPTSNH	LPTSCPPTCP	GYRWMCLRRF	IIFLFILLLC	LIFLLVLLDY	QGMLPVCPLL	PGSTTTSTGP
F2	MENITSGLLG	PLLVLQAVCF	LLTKILTIPQ	SLDSWWTSLN	FLGGLPGCPG	QNSQSPTSNH	LPTSCPPTCP	GYRWMCLRRF	IIFLFILLLC	LIFLLVLLDY	QGMLPVCPLL	PGSTTTSTGP
F3	MDNITSGLLG	PLLVLQAVCF	LLTKILTIPQ	SLDSWWTSLN	FLGGLPGCPG	QNSQSPTSNH	LPTSCPPTCP	GYRWMCLRRF	IIFLFILLLC	LIFLLVLLDY	QGMLPVCPLI	PGSTTTSTGP
G1	MENITSGFLG	PLLVLQAGFF	LLTRILTIPQ	SLDSWWTSLN	FLGGVPVCPG	LNSQSPTSNH	SPISCPPTCP	GYRWMCLRRF	IIFLFILLLC	LIFLLVLLDY	QGMLPVCPLI	PGSSTTSTGP
G2	MENITSGFLG	PLLVLQAGFF	LLTRILTIPQ	SLDSWWTSLN	FLGGVPVCPG	LNSQSPTSNH	SPISCPPTCP	GYRWMCLRRF	IIFLFILLLC	LIFLLVLLDY	QGMLPVCPLI	PGSSTTSTGP
H1	MENITSGLLG	PLLVLQAVCF	LLTKILTIPQ	SLDSWWTSLN	FLGVPPGCPG	QNSQSPISNH	LPTSCPPTCP	GYRWMCLRRF	IIFLFILLLC	LIFLLVLLDY	QGMLPVCPLL	PGSTTTSTGP
H2	MENITSGLLG	PLLVLQAVCF	LLTKILTIPK	SLDSWWTSLN	FLGVPPGCPG	QNSQSPISNH	LPTSCPPTCP	GYRWMCLRRF	IIFLFILLLC	LIFLLVLLDY	QGMLPVCPLL	PGSTTTSTGP
H3	MENITSGLLG	PLLVLQAVCF	LLTKILTIPQ	SLDSWWTSLN	FLGVPPGCPG	QNSQSPISNH	LPTSCPPTCP	GYRWMCLRRF	IIFLFILLLC	LIFLLVLLDY	QGMLPVCPLL	PGSTTTSTGP
I1	MENITSGFLG	PLLVLQAGFF	LLTKILTIPQ	SLDSWWTSLN	FLGGSTVCLG	QNSQSPTSNH	SPTSCPPICP	GYRWMCLRRF	IIFLFILLLC	LIFLLVLLDY	QGMLPVCPLI	PGSSTTSTGP
I2	MENITSGFLG	PLLVLQAGFF	LLTKILTIPQ	SLDSWWTSLN	FLGGAPVCLG	QNSQSPTSNH	SPTSCPPICP	GYRWMCLRRF	IIFLFILLLC	LIFLLVLLDY	QGMLPVCPLI	PGSSTTSTGP
J1	MENITSGFLG	PLLVLQAGFF	LLTKILTIPQ	SLDSWWTSLN	FLGGALVCPG	QNSQSLTSNH	SPTSCPPTCP	GYRWMCLRRF	IIFLFILLLC	LIFLLVLLDY	QGMLPVCPLL	PGSTTTSTGP
ayr1	MERQTSGFLG	PLLVLQAGFF	LLTRILTIPQ	SLDSWWTSLN	FLGGAPTCPG	QNSQSPTSNH	SPTSCPPICP	GYRWMCLRRF	IIFLFILLLC	LIFLLVLLDY	QGMLPVCPLL	PGTSTTSTGP
WT	MENITSGFLG	PLLVLQAGFF	LLTRILTIPQ	SLDSWWTSLN	FLGGTTVCLG	QNSQSPTSNH	SPTSCPPTCP	GYRWMCLRRF	IIFLFILLLC	LIFLLVLLDY	QGMLPVCPLI	PGSSTTSTGP

	121 130	140	150	160	170	180	190	200	210	220	226	Variation (%)
A1	CKTCTTPAQG	NSMFPSCCCT	KPTDGNCTCI	PIPSSWAFAK	YLWEWASVRF	SWLSLLVPFV	QWFVGLSPTV	WLSAIWMMWY	WGPSLYSIVS	PFIPLLPIFF	CLWVYI	7.1
A2	CKTCTTPAQG	NSMFPSCCCT	KPTDGNCTCI	PIPSSWAFAK	YLWEWASVRF	SWLSLLVPFV	QWFVGLSPTV	WLSVIWMMWY	WGPSLYNILS	PFIPLLPIFF	CLWVYI	6.6
B1	CKTCTTPAQG	TSMFPSCCCT	KPTDGNCTCI	PIPSSWAFAK	YLWEWASVRF	SWLSLLVPFV	QWFVGLSPTV	WLSVIWMMWF	WGPSLYNILS	PFMPLLPIFF	CLWVYI	9.7
B2	CKTCTTPAQG	TSMFPSCCCT	KPTDGNCTCI	PIPSSWAFAK	YLWEWASVRF	SWLSLLVPFV	QWFVGLSPTV	WLSVIWMMWF	WGPSLYNILS	PFIPLLPIFF	CLWVYI	10.2
C1	CKTCTIPAQG	TSMFPSCCCT	KPSDGNCTCI	PIPSSWAFAR	FLWEWASVRF	SWLSLLVPFV	QWFVGLSPTV	WLSVIWMMWY	WGPSLYNILS	PFLPLLPIFF	CLWVYI	7.5
C2	CKTCTTPAQG	TSMFPSCCCT	KPSDGNCTCI	PIPSSWAFAR	FLWEWASVRF	SWLSLLVPFV	QWFVGLSPTV	WLSVIWMMWY	WGPSLYNILN	PFLPLLPIFF	CLWVYI	8.4
D1	CRTCTTPAQG	TSMYPSCCCT	KPSDGNCTCI	PIPSSWAFGK	FLWEWASARF	SWLSLLVPFV	QWFVGLSPTV	WLSVIWMMWY	WGPSLYSILS	PFLPLLPIFF	CLWVYI	1.8
D2	CRTCTTTVQG	TSMYPSCCCT	KPSDGNCTCI	PIPSSWAFGK	FLWEWASARF	SWLSLLVPFV	QWFVGLSPTV	WLSVIWMMWY	WGPRLYSILS	PFLPLLPIFF	CLWVYI	2.7
E1	CRTCTTLAQG	TSMFPSCCCS	KPSDGNCTCI	PIPSSWAFGK	FLWEWASARF	SWLSLLVPFV	QWFAGLSPTV	WLSVIWMMWY	WGPSLYNILS	PFIPLLPIFF	CLWVYI	5.3
E2	CRTCTTLAQG	TSMFPSCCCS	KPSDGNCTCI	PIPSSWAFGK	FLWEWASARF	SWLSLLVPFV	QWFAGLSPTV	WLSVIWMMWY	WGPSLYNILS	PFIPLLPIFF	CLWVYI	5.8
F1	CKTCTTLAQG	TSMFPSCCCS	KPSDGNCTCI	PIPSSWALGK	YLWEWASARF	SWLSLLVQFV	QWCVGLSPTV	WLLVIWMIWY	WGPNLCSILS	PFIPLLPIFC	YLWVSI	11.9
F2	CKTCTTLAQG	TSMFPSCCCS	KPSDGNCTCI	PIPSSWALGK	YLWEWASARF	SWLSLLVQFV	QWCVGLSPTV	WLLVIWMIWY	WGPNLCSILS	PFIPLLPIFC	YLWVSI	11.5
F3	CKTCTTLAQG	TSMFPSCCCS	KPSDGNCTCI	PIPSSWALGK	YLWEWASARF	SWLSLLVQFV	QWCVGLSPTV	WLLVIWMIWY	WGPNLCSILS	PFIPLLPIFC	YLWVSI	11.5
G1	CKTCTTPAQG	NSMYPSCCCT	KPSDGNCTCI	PIPSSWAFAK	YLWEWASVRF	SWLSLLVPFV	QWFVGLSPTV	WLSAIWMMWY	WGPNLYNILS	PFIPLLPIFF	CLWVYI	6.2
G2	CKTCTTPAQG	NSMYPSCCCT	KPSDGNCTCI	PIPSSWAFAK	YLWEWASVRF	SWLSLLVPFV	QWFVGLSPTV	WLSVIWMMWY	WGPNLYNILS	PFIPLLPIFF	CLWVYI	5.8
H1	CKTCTTLAQG	TSMFPSCCCT	KPSDGNCTCI	PIPSSWAFGK	YLWEWASARF	SWLSLLVQFV	QWCVGLSPTV	WLLVIWMIWY	WGPNLCSILS	PFIPLLPIFC	YLWASI	11.9
H2	CKTCTTLAQG	TSMFPSCCCT	KPSDGNCTCI	PIPSSWAFGK	YLWEWASARF	SWLSLLVQFV	QWCVGLSPTV	WLLVIWMIWY	WGPNLCSILS	PFIPLLPIFC	YLWASI	12.4
H3	CKTCTTLAQG	TSMFPSCCCT	KPSDGNCTCI	PIPSSWAFGK	YLWEWASARF	SWLSLLVQFA	QWCVGLSPTV	WLLVIWMIWY	WGPNLCSILS	PFIPLLPIFC	YLWASI	12.4
I1	CRTCTTPAQG	NSMYPSCCCT	KPSDGNCTCI	PIPSSWAFAK	YLWEWASARF	SWLSLLVPFV	QWFVGLSPTV	WLSVIWMMWY	WGPSLYNILS	PFIPLLPIFF	CLWVYI	4.4
I2	CKTCTTPAQG	NSMYPSCCCT	KPSDGNCTCI	PIPSSWAFAK	YLWEWASARF	SWLSLLVPFV	QWFVGLSPTV	WLSVIWMMWY	WGPSLYNILS	PFIPLLPIFF	CLWVYI	5.3
J1	CRTCTITAQG	TSMFPSCCCT	KPSDGNCTCI	PIPSSWAFAK	FLWEWASVRF	SWLSLLAPFV	QWFAGLSPTV	WLSVIWMIWY	WGPSLYNILN	PFIPLLPIFF	CLWVYI	7.1
ayr1	CRTCTTPAQG	TSMFPSCCCT	KPSDGNCTCI	PIPSSWAFAR	YLWEWASFRF	SWLSLLVPFV	QWFVWLSPTV	WLSVIWMMWY	WGPSLYNILS	PFIPLLPIFL	CLWVYI	8.0
WT	CRTCMTTAQG	TSMYPSCCCT	KPSDGNCTCI	PIPSSWAFGK	FLWEWASARF	SWLSLLVPFV	QWFVGLSPTV	WLSVIWMMWY	WGPSLYSILS	PFLPLLPIFF	CLWVYI	0.0

Supporting Information 2

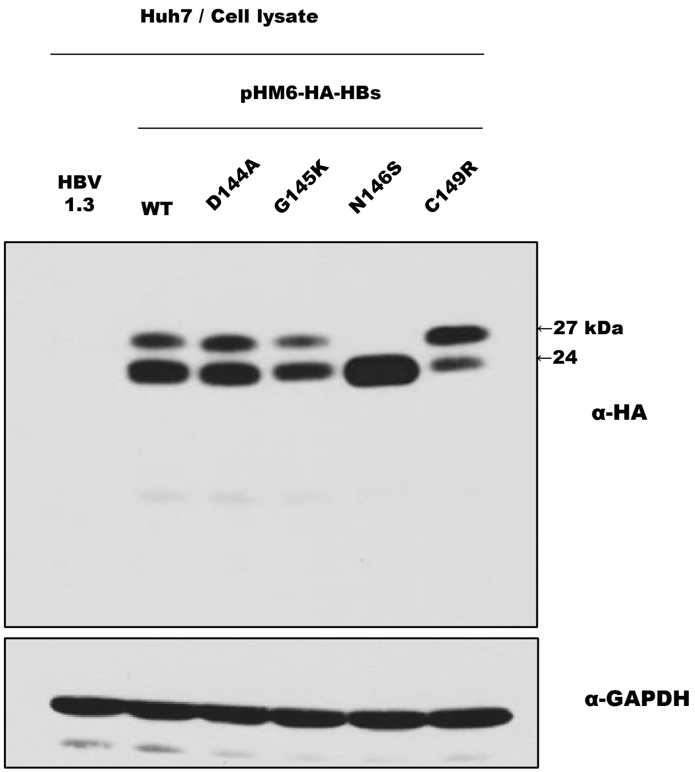

SDS-PAGE (15%)

Original images of figure 1

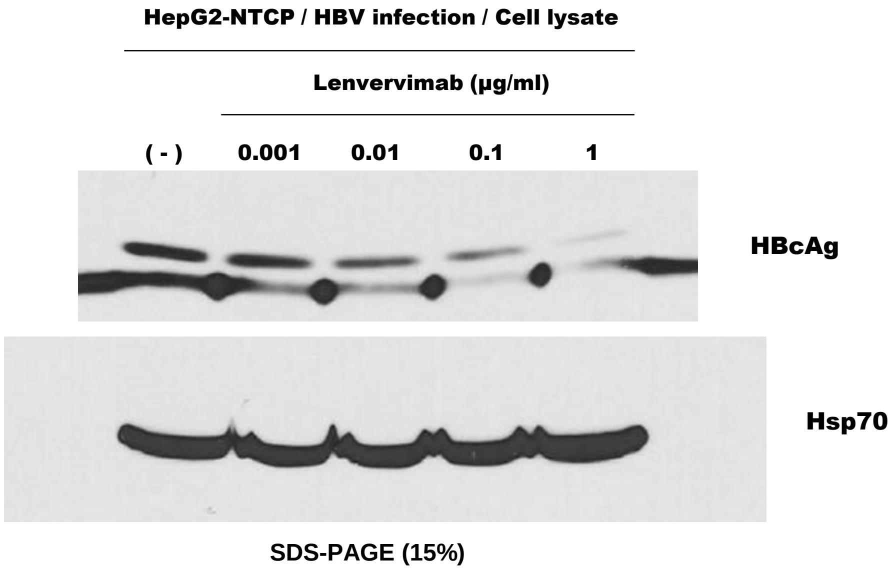

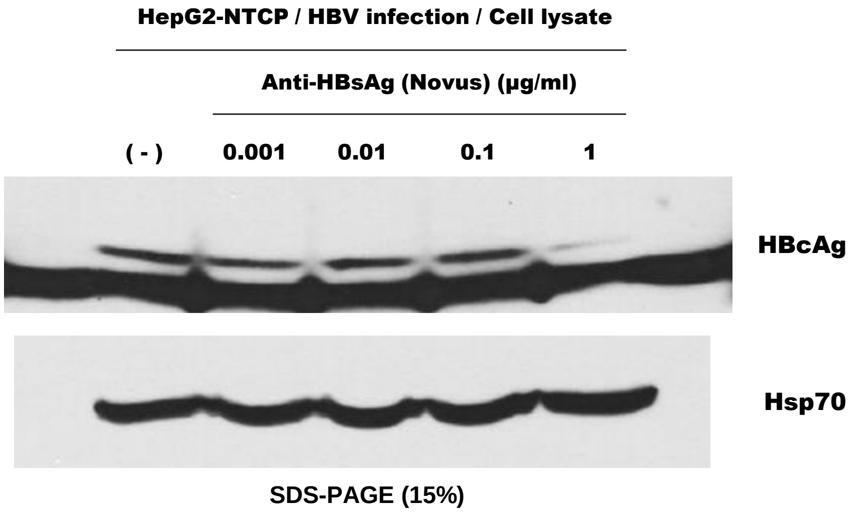

Original images of figure 2

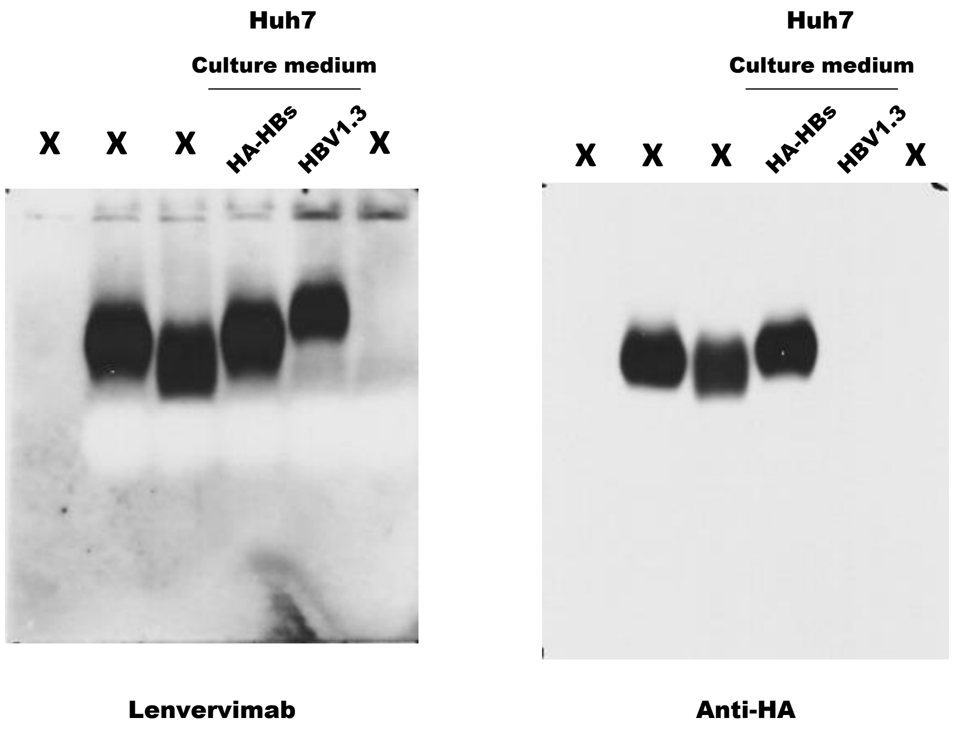

Native agarose gel (1%)

Original images of figure 2

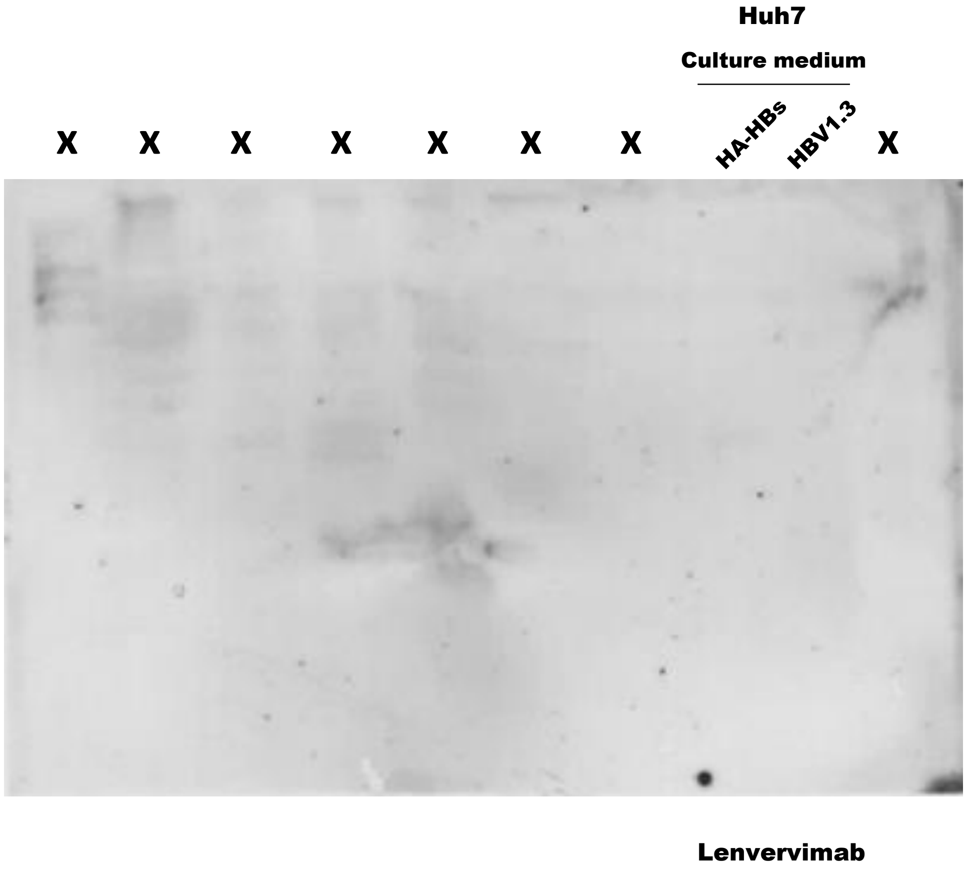

Original images of figure 2

Huh7

Culture medium

X X X X X X X HA-HBs HBV1.3 X

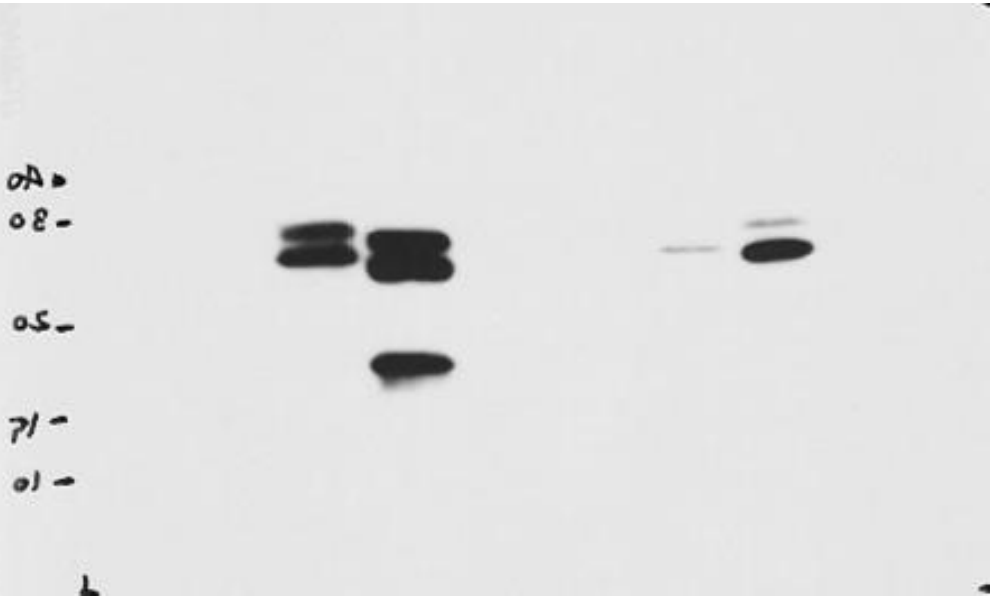

Anti-HA

SDS-PAGE (15%)

Original images of figure 3A

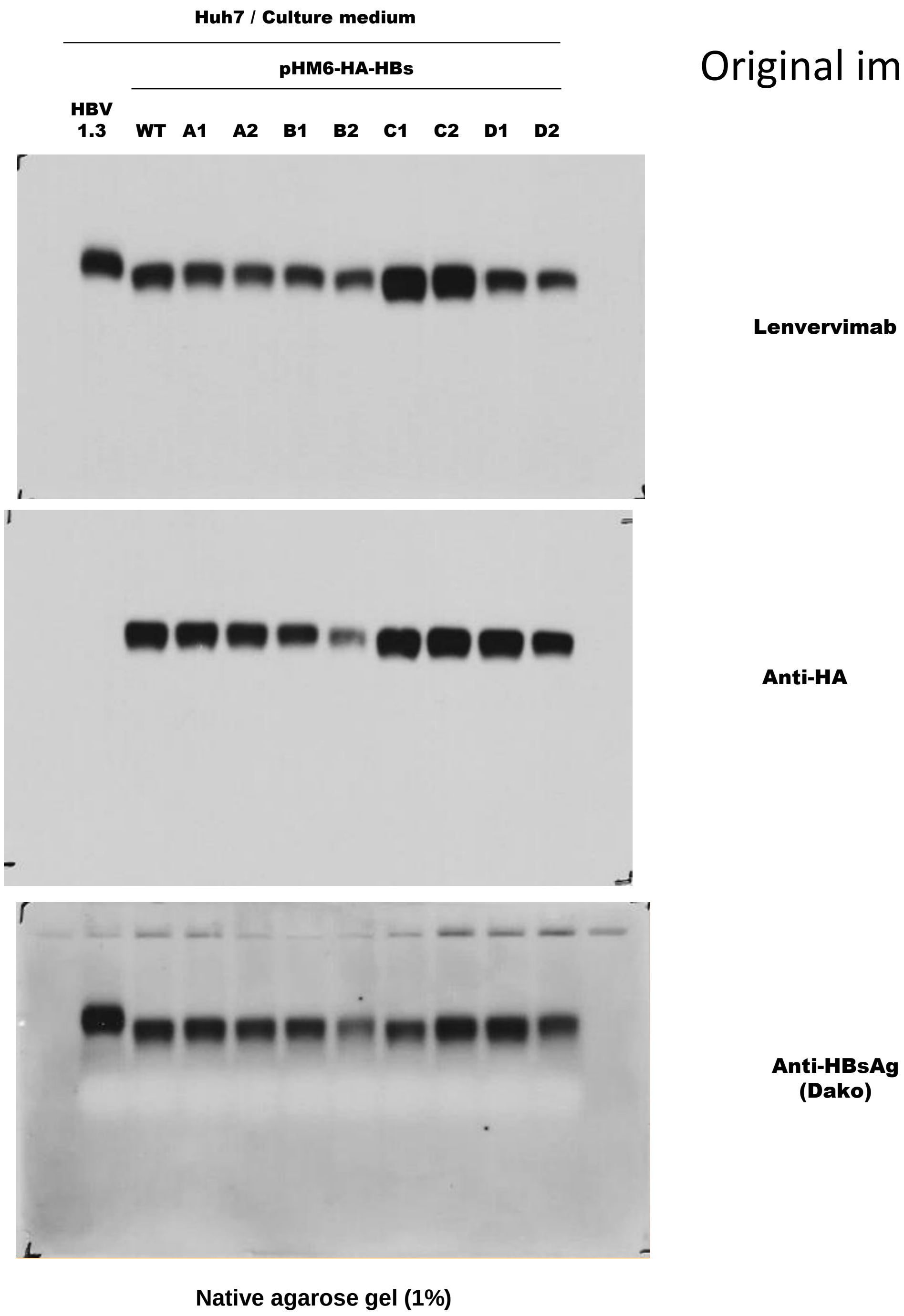

Original images of figure 3A

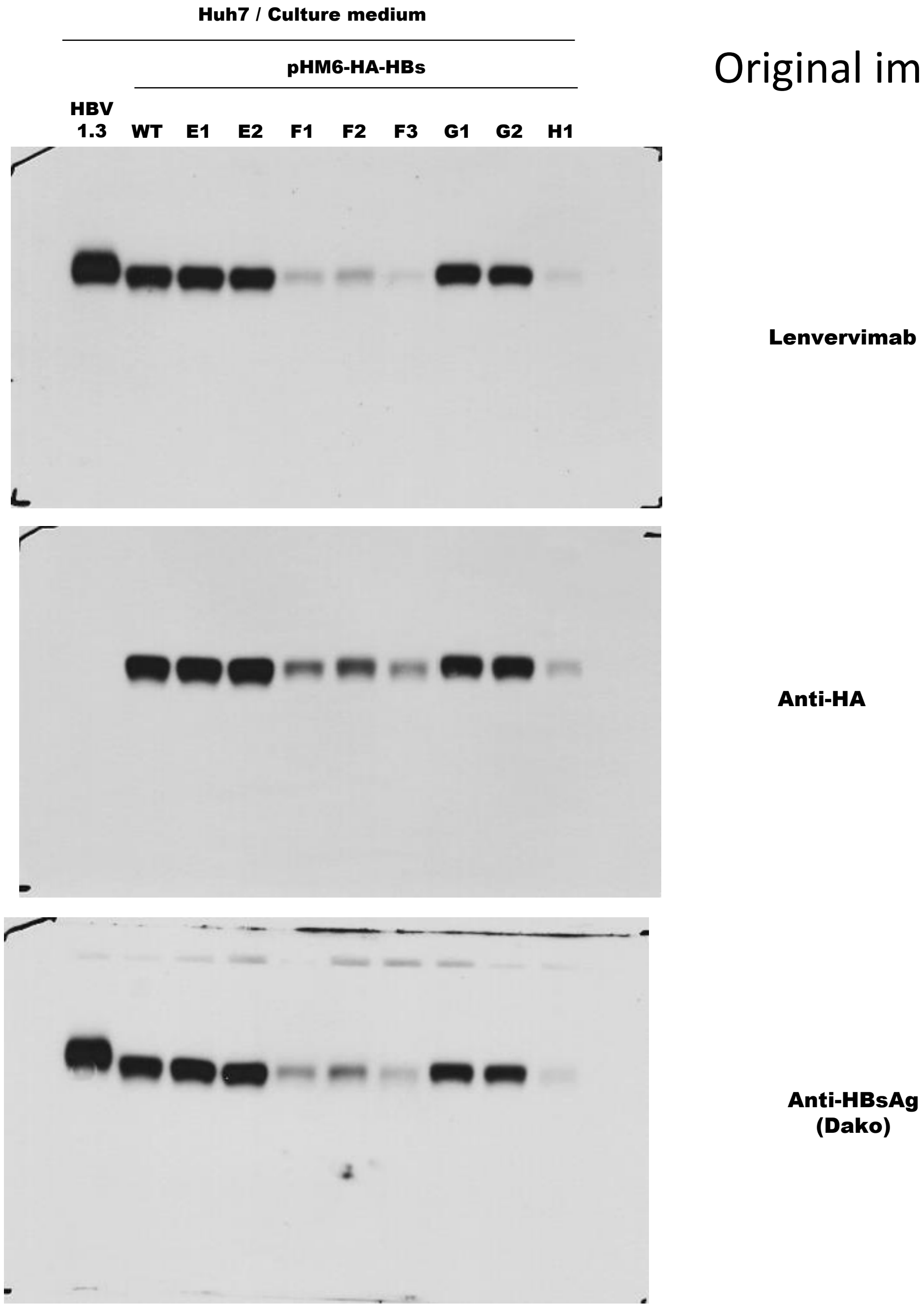

Original images of figure 3A

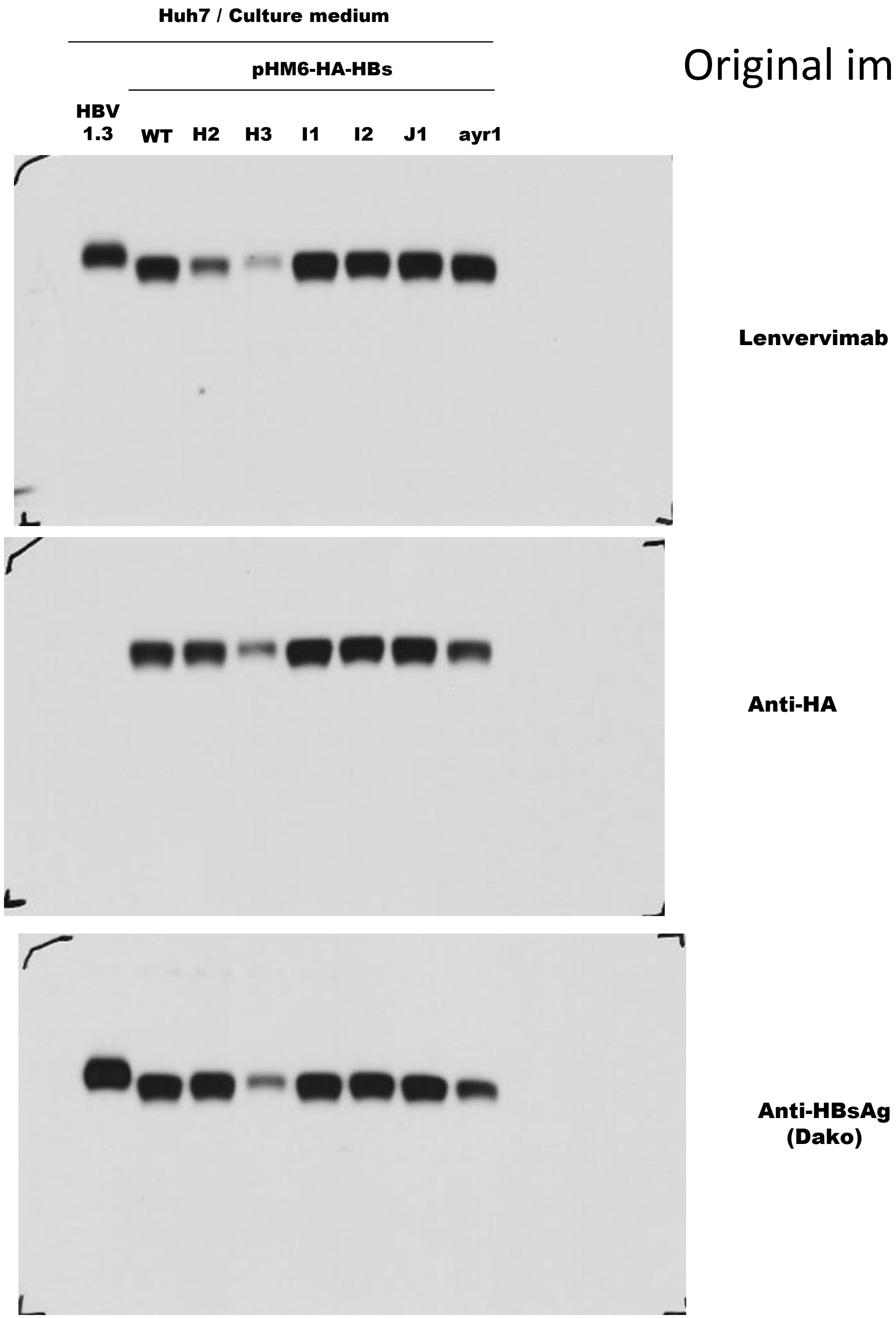

Original images of figure 5A

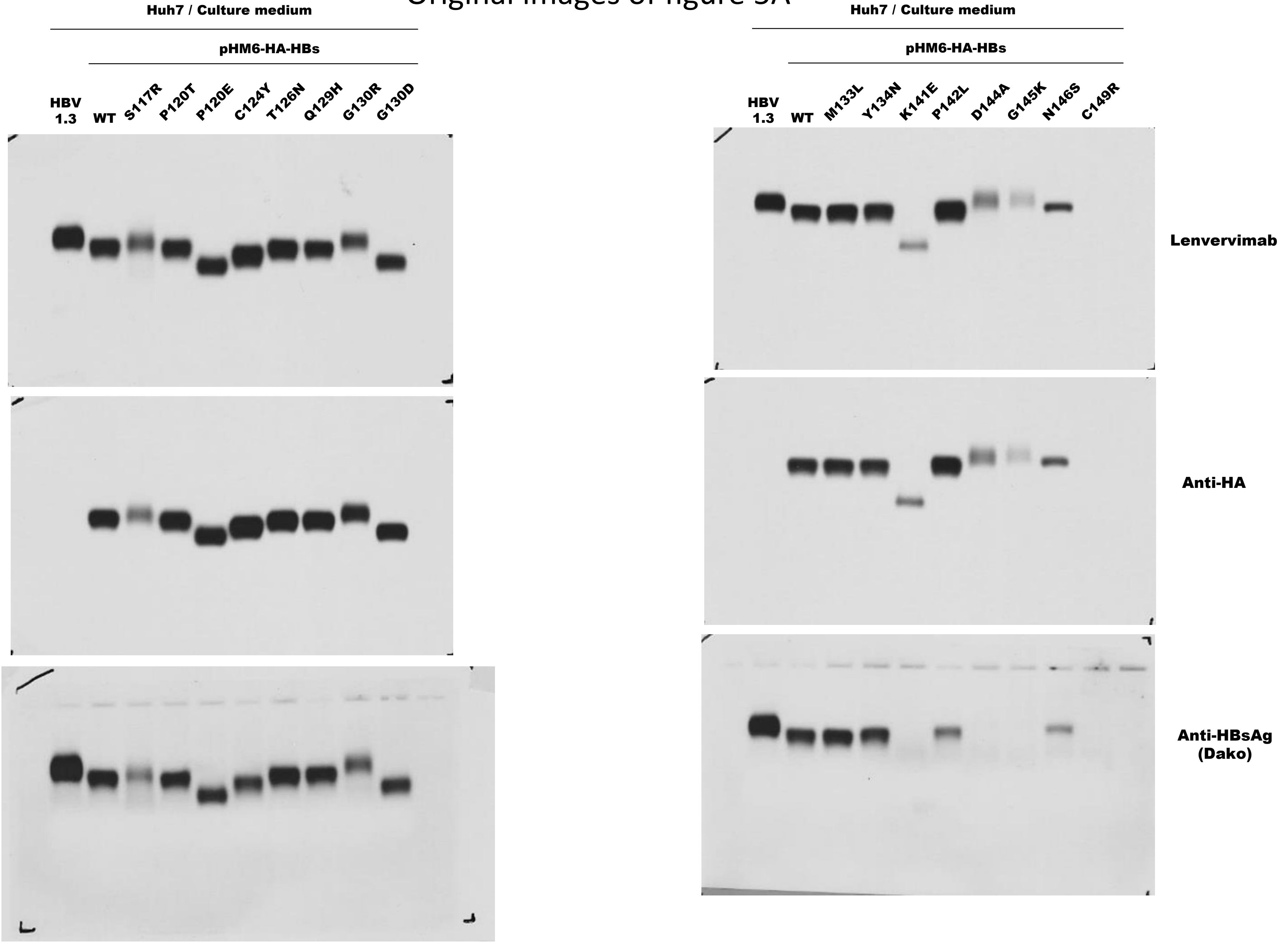

Native agarose gel (1%)

Original images of figure 5B

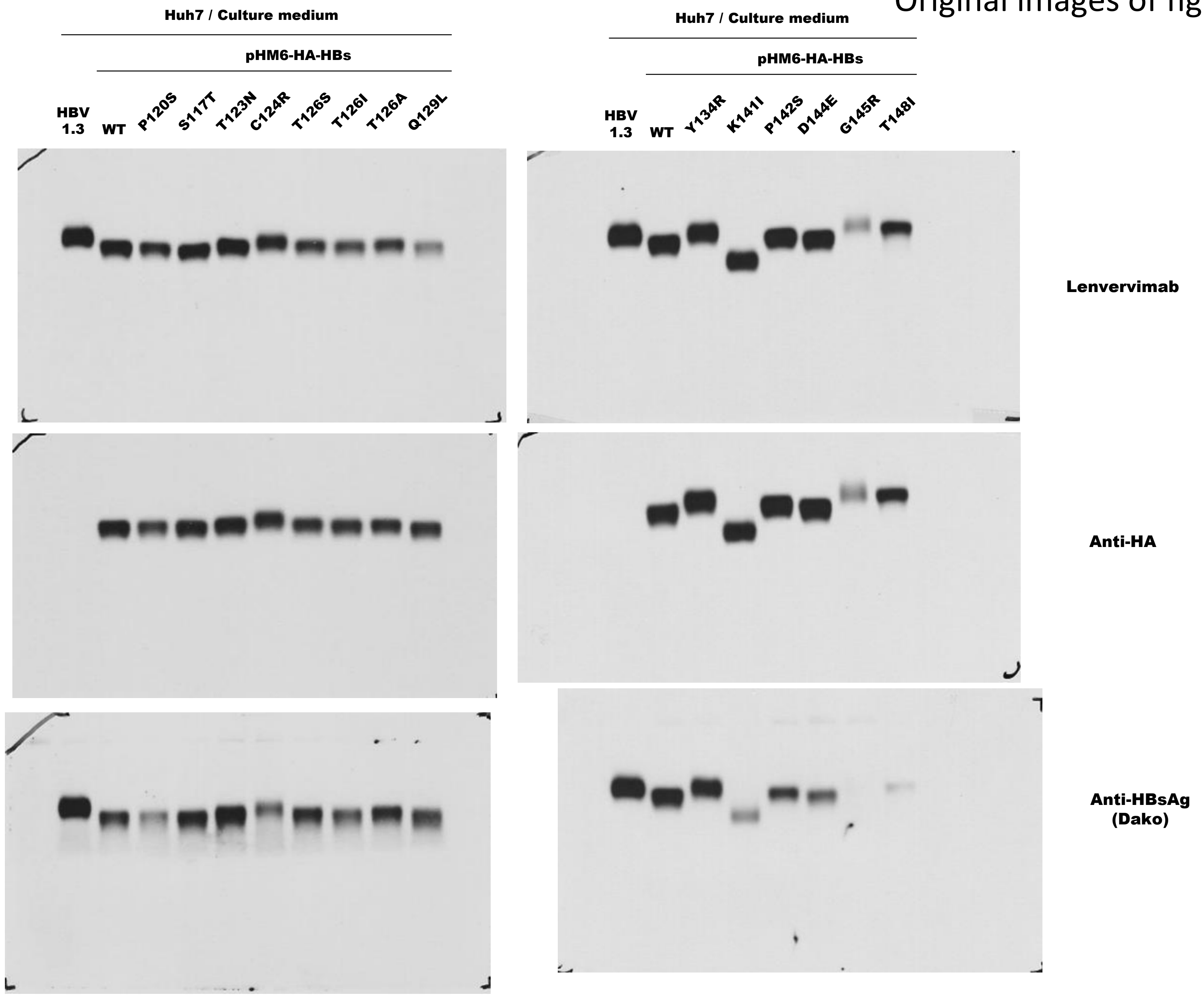

Native agarose gel (1%)

Original images of figure 6A

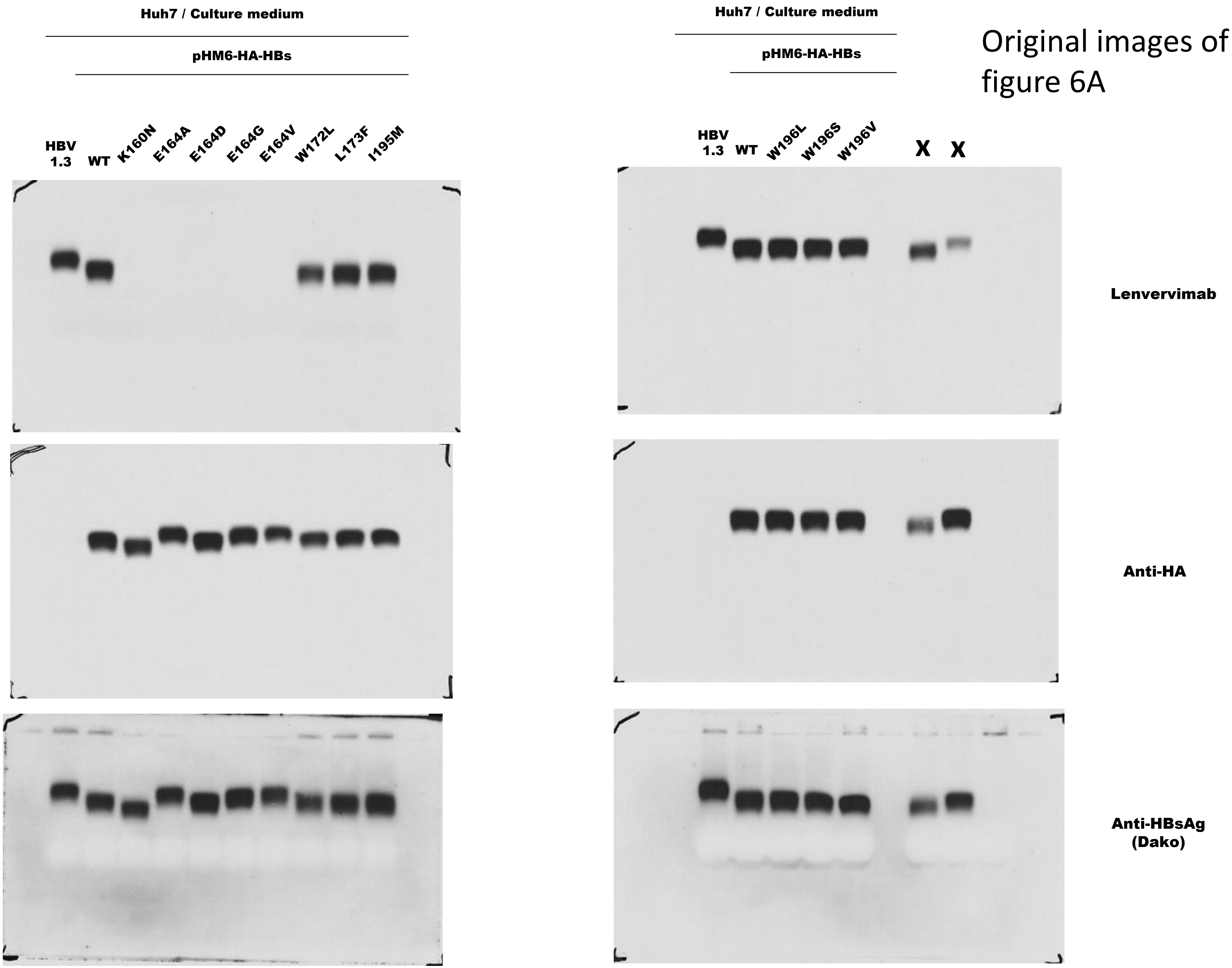

Native agarose gel (1%)

Original images of figure 6A

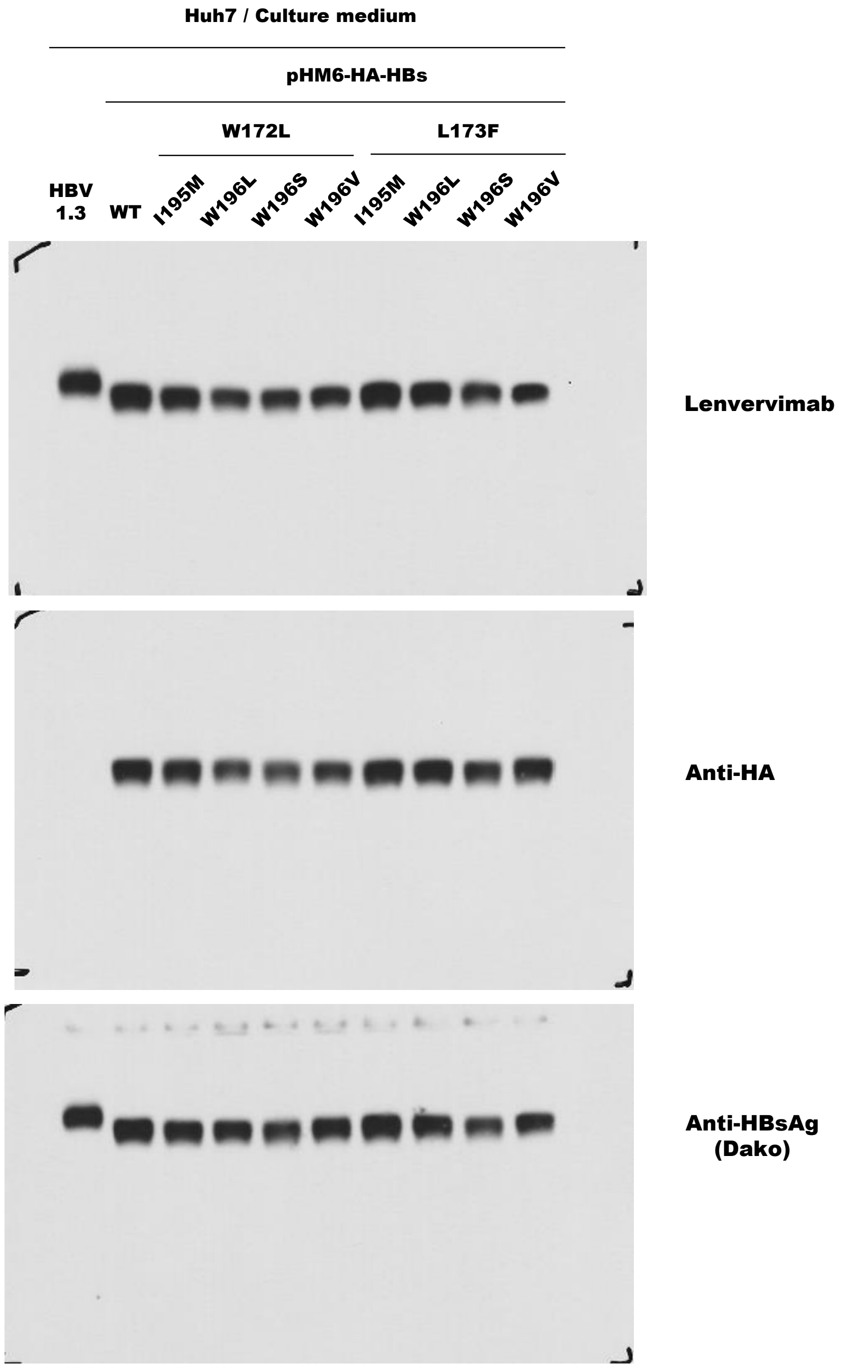

Original images of Supporting Info.

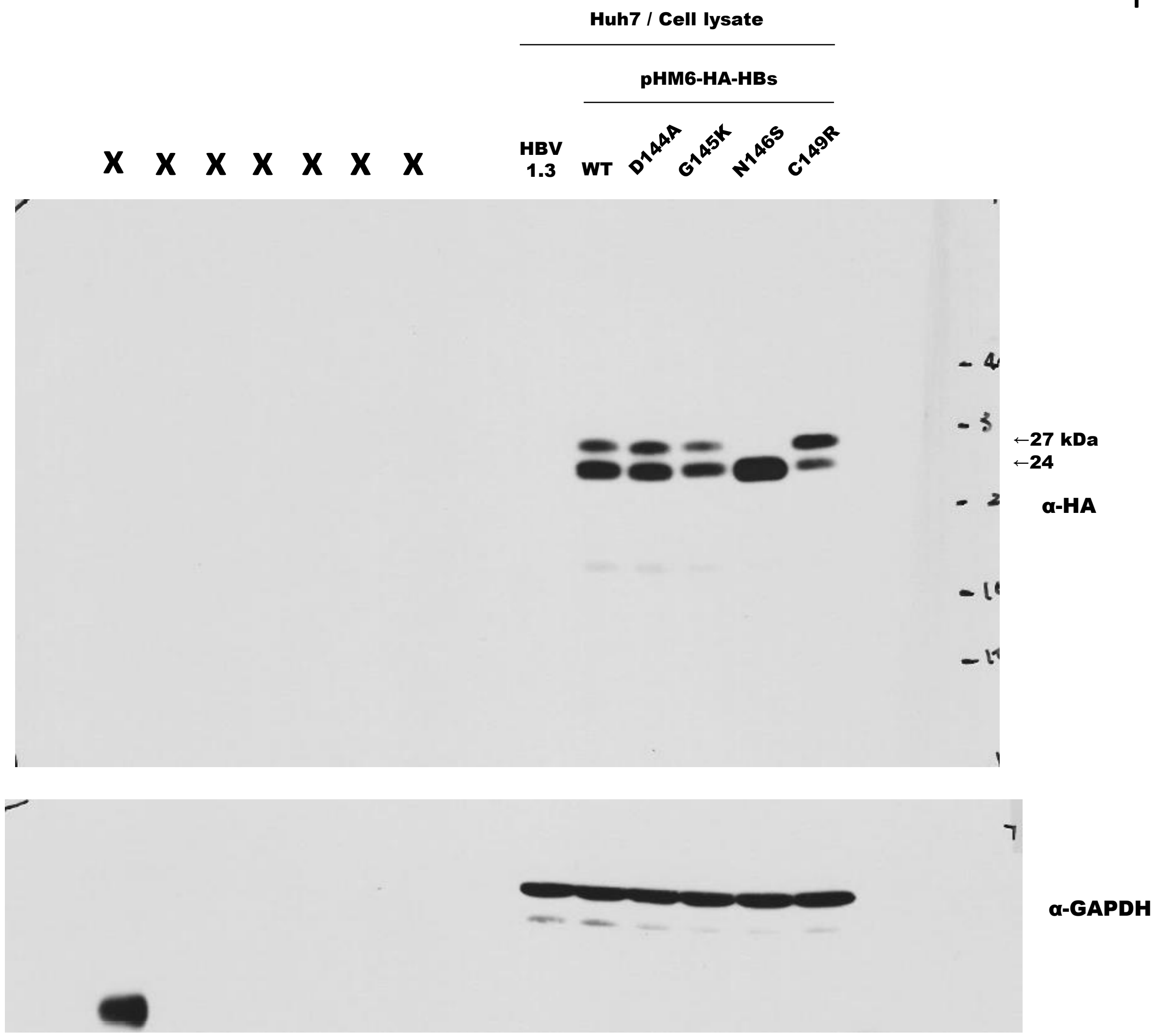

SDS-PAGE (15%)
